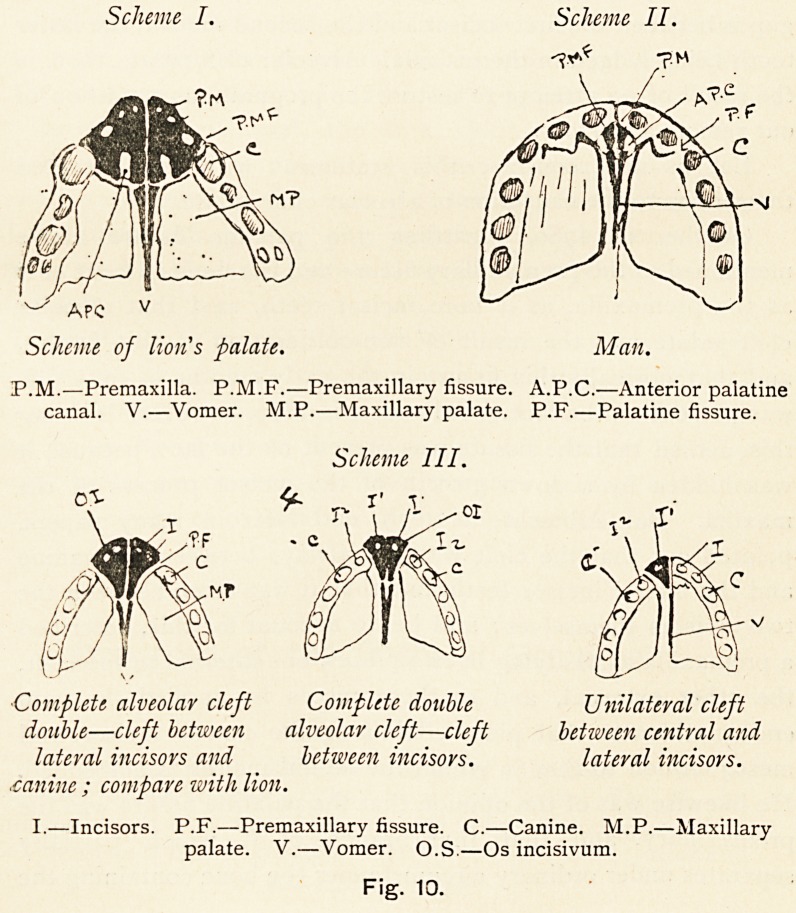# The Explanation of Alveolar Cleft Palate

**Published:** 1906-09

**Authors:** Edward Fawcett

**Affiliations:** Professor of Anatomy in University College, Bristol


### THE EXPLANATION OF ALVEOLAR CLEFT PALATE.

BY

EDWARD FAWCETT, M.D. Edin.,

*Professor of Anatomy in University College, Bristol.*

THE various theories which have been propounded to account for the different forms of alveolar cleft palate all rest on the same foundation, which is the assumption that certain fissures to be seen normally on all young human palates are indications that the superior maxillary bone developmentally consists of two morphologically different elements, viz. the maxilla, which bears the canine and molar teeth, and the premaxilla, in which the incisors are lodged. And these theories, moreover, assume that in cleft palate of the alveolar form these fissures, which they claim are complete in early life, remain so after birth, and constitute the cleft or clefts, as the case may be. Furthermore, to strengthen the argument, they assert that these palatine fissures are homologous with those found between the premaxillæ and maxillæ in the lower animals, and so name them premaxillary fissures. Finally, to clench the argument, they assume that because the bone in front of these fissures bears the incisor teeth in lower mammals and is premaxilla, the part of the human maxilla in front of these fissures is also premaxilla, and that given non-union of these parts developmentally, alveolar cleft palate is the result.

If it can be shown that these palatine fissures of man are not homologous with the premaxillary fissure of lower animals, and, further, that the incisor teeth are not normally borne by the premaxilla but by the maxilla, then these theories fall to the ground.

The aim of this paper is to show that neither the above-mentioned fissures nor the incisor-bearing bones are homologous, and to do that, evidence must be sought from embryology and from comparative anatomy. But before adducing this evidence, it will be well to examine both the fissures and the bones as they exist normally in man and the lower animals.

1. *The Fissures.*

(*a*) *In man.*—We find on the under aspect of the bony palate, immediately behind the suture between the two central incisor teeth, a fossa—the anterior palatine fossa, which is seen to lead into two canals—the anterior palatine canals. Each canal is separated from its fellow by two narrow bars of bone, which are held together by a suture. Passing outwards from the middle of the lateral walls of the fossa is a fissure; this fissure, after indenting the bone between the central and lateral incisor teeth, terminates externally at the front of the *inner* alveolar wall of the canine tooth, and it never appears on the

Fig. 1.

*The human palate at five years, showing the completion of the palate and palatine fissures.*

face. This fissure has been assumed to be *the* premaxillary fissure, and it lies behind the incisor teeth.

(*b*) *In lower animals.*—In the lion no anterior palatine fossa is seen, but two large anterior palatine canals are present, separated by two narrow bars of bone as in man, and from these canals there pass outward on their respective sides fissures, and these fissures not only traverse the inner alveolar wall of the canine tooth, *but the outer one as well, and appear on the face.* From the fact that they run out from the anterior palatine canals to

Fig. 2.

*Palate of lion from below.*

I.—Incisors. C.—Canine. P.M.—Premaxilla. P.P.—Palatine process of premaxilla. P.M.F.—Premaxillary fissure.

the front of the canine tooth, they are assumed to be homologous with the so-called premaxillary fissures of man, and the bone lying in front bearing the incisor teeth is assumed to be homologous with that part which bears the incisor teeth in man.

2. *The Bones which bear the incisor teeth.*

In man these bones are seen in front of the palatine fissure and seem to consist of two segments, as indicated by their partial separation by the extension into them of branches of the palatine fissure, one such extension being between the two incisor teeth, the other being between the lateral incisor and the canine (Fig. 1); but complete separation is never seen, as these branches of the palatine fissure never extend through the outer alveolar wall to the face. The incisor-bearing bone then in man is directly continuous with the upper jaw.

In the lion the incisor-bearing bone is completely cut off from the maxilla by a fissure which extends to the face between the lateral incisor and the canine tooth. This incisor-bearing bone viewed from the under aspect consists of an alveolo-facial mass (P.M., Fig. 2), which sends backwards, on the inner side of the anterior palatine canal, a process—the palatine process (P.P., Fig. 2); this process articulates with its fellow mesially, with the

Fig. 3.

*Forepart of lion's skull from above.*

V.—Vomer. M.—Maxilla. P.M.—Premaxilla. P.P.—Palatine process of premaxilla. A.P.C.—Anterior palatine canal. P.M.F.—Premaxillary fissure.

palatine process of the maxilla behind and with the vomer above.

When these bones are viewed from above, we notice in the lion that the most anterior part of the skull is formed by the alveolo-facial part (P.M., Fig. 3) of the incisor tooth-bearing bone, the premaxilla, and that this bone is separated by a fissure (P.M.F.) from the maxilla proper. We notice too the spur of bone (P.P.) projected backwards in the floor of the nose, to end under the anterior extremity of the vomer (V.), and that it bounds mesially the anterior palatine canal (A.P.C.) of its side. In man, from the same point of view, we see the incisor tooth-bearing mass on the face also in the floor of the nose, but it is not separated on the face by a fissure from that bone which bears the canine and molar teeth. We see too the anterior palatine canals bounded mesially by two small bars of bone (P.M.), which together help to form the inferior nasal crests, and which articulate behind and above with the vomer. These bars have hitherto been regarded as parts of the superior maxilla. They are best seen

Fig. 4.

*The Maxillæ* (M.), *the Premaxillæ* (P.M.), *and Vomer* (V.), *from above and in front.*

I.—Incisors. C.—Canine. A.P.C.—Ant. Palatine Canal.

from above, and although visible from below they do not enter into the oral surface of the bony palate, but lie deeply in the anterior palatine fossa (P.M., Fig. 1). We will return to them later.

We will now discuss the embryology of these parts.

*Embryological evidence.*

It is well known that the face—bones and soft tissues—is formed around the primitive mouth, formed by the appearance, growth and approximation of certain processes known as the fronto-nasal, maxillary and mandibular. The fronto-nasal process lies above and forms the upper boundary; the mandibular, two in number, meet below the primitive mouth, and

Fig. 5.

*Formation of face.*

from their dorsal ends the maxillary process grow into the cleft between the mandibular arches and the fronto-nasal process on each side (Fig. 5).

Each of these processes consists of a superficial element which helps to form the lip—the labial element, and a deep element in which bones are formed—the osteogenetic element,

Fig. 6.

*View of the upper boundaries of the primitive mouth from below in lower animals and in the early human embryo.*

as can be seen when the mandibular arches are removed and the roof of the primitive mouth viewed (Fig. 6).

In the lower animals and in man the labial elements of the above-named processes fuse with one another to form the lip, failure of fusion producing harelip. But the osteogenetic elements behave quite differently in lower animals and in man. In lower animals the osteogenetic element of the fronto-nasal process gives rise in course of time to the premaxillæ and the vomer, both being at first paired; the osteogenetic element of the maxillary process becomes ossified to form at least the maxilla. The premaxillæ enlarge in an outward and backward direction, and meet the forwardly-growing maxillæ at a suture—the premaxillary suture which is immediately behind the fissure in the lip. This condition is the permanent one in lower animals, only being somewhat modified by the ingrowth of the palatine processes of the superior maxillary bone.

In man at first the same conditions are seen as figured in Figure 6, but as development proceeds there appear those changes which place man apart from the lower animals. The osteogenetic elements of the maxillary processes grow forwards and inwards underneath the corresponding element of the fronto-nasal process and fuse in the middle line, shutting out from the mouth the premaxillary *anlagen* in the fronto-nasal process.

Fig. 7.

*The growth forwards of the incisor process of the maxilla characteristic of man.*

Later, the bony maxilla extends in the osteogenetic element of the maxillary process to the middle line as well, and in it are developed the incisor teeth. The premaxilla in man, therefore, loses its incisor tooth-bearing function; and, moreover, because the maxilla has grown in underneath it, as well as in front of it, the premaxilla is not only excluded from the roof of the mouth, but also from the face, and is visible as a small bar-like bone in the nasal septum. Here, then, is direct evidence that the bone which in man bears the incisor teeth is not premaxilla, and cannot be therefore homologous with the premaxilla of lower animals which does bear these teeth. But this is only schematic evidence, as the drawing is a scheme based on sectional anatomy of the young embryo.

If we make serial sections of a young embryo, and the one presented is a coronal section of a "Minot" embryo of 19 mm. in length, we actually see these things which have been previously schematised.

The nasal septum (N.S.) (fronto-nasal process, median part) is seen separating the two nasal cavities (N.C.); in its middle is a

Fig. 8.

*Coronal section of "Minot" embryo of 19 mm.*

cartilaginous skeleton ending below in a bulb, on each side of which are the Jacobsonian organs (J.O.), and depending from this bulb are the two Jacobsonian cartilages (J.C.), in the area between these are the *anlagen* of the premaxillary bones. The osteogenetic element of the maxillary process (o.) is seen fusing with the outer side of the fronto-nasal process, but not in its whole height; a great part of the maxillary process is preparing to pass beneath the fronto-nasal process to meet its fellow in the middle line as indicated by the arrows. By meeting underneath the fronto-nasal processes and the continued *anlagen* of the premaxillary bones, these bones become shut out from the mouth. On one side of the section the nasal cavity is seen to run into the mouth, that communication becomes subsequently the anterior palatine canal, and it is important to notice that the premaxillary *anlage* lies to its mesial side, a position which it always retains. In the mesoblast (M.) of the osteogenetic segment (o.) of the maxillary process the incisor bony process will be developed in continuity with the rest of the maxilla, and underneath this mesoblast an incisor tooth germ can be seen. No such tooth germ (T.) can be seen in relation to the fronto-nasal process and its contained premaxilla.

The failure of the fronto-nasal process to reach downwards to the level of the under margin of the osteogenetic element of the maxillary processes, and thus to bring the premaxillary bones into the oral surface of the roof of the mouth, *is the essential difference between man and the lower animals.*

Now let us return to the further growth of that part of the bony maxilla which grows under the premaxilla, as the incisor process or incisor tooth-bearing part of the maxilla. Before doing so, it may perhaps be well to state that by this time the part of the maxilla containing the canine and molar teeth (A.M.P.) has begun to send inwards in the soft tissues of the hard palate a shelf-like growth, which is a part of the permanent bony palate, and soon assumes a triangular form, with opening backwards. The incisor process (I.P.) follows suit, sending backwards two processes, one behind the central incisor, the other behind the lateral one, and in order to accommodate themselves to the space which they occupy they are wedge-shaped in form. As they grow they approach one another and the anterior border of the triangular palatine plate which has preceded them in growth, and it is thus that the palatine fissure (P.F.) is produced which previously has always been regarded as the premaxillary fissure; at its inner end it ends in a vacuity—the anterior palatine canal. This palatine fissure then, from the mode of its development, cannot be homologous with the premaxillary fissure of

Fig. 9.

*Scheme of the formation of the palatine fissures.*

animals, because it is formed entirely in the maxilla, and does not separate maxilla from premaxilla. From the nature of its formation it cannot run out to the face nor can any of its branches.

Where, then, is the premaxillary fissure? Where is the premaxilla? And why is it, when located, where it is?

The premaxilla in man is a small bone, which is generally described as a part of the superior nasal crest of the superior maxilla (P.M., Fig. 4), and which articulates above with the vomer by its anterior end; it is fused in later life with the maxilla. By its posterior end it projects freely backwards to the mesial side of the upper end of the anterior palatine canal, and articulates with the under border of the anterior end of the vomer (V.). In its relations, as seen from above, it is identical with that process of the premaxilla which described previously in the lion's skull as passing backwards from the alveolo-facial mass, bounded mesially by the anterior palatine canal of its side, and projected under the anterior end of the vomer (P.P., Fig. 3).

In man it is only visible from below when the anterior palatine fossa is looked into, and its bounding fissure is also to be seen in that fossa opening into the anterior palatine canal; but not reaching the face, the premaxilla is where it is, and is toothless because, as has been shown developmentally, it is shut out by the growth beneath and in front of it of the superior maxilla, and that growth is the result of the change from prognathism to orthognathism. In the truly prognathous skull the premaxilla is the most anterior bone of the face; in the orthognathous skull, as in man, the superior maxilla is. But

*Scheme I.*

*Scheme of lion's palate.*

*Scheme II.*

*Man.*

P.M.—Premaxilla. P.M.F.—Premaxillary fissure. A.P.C.—Anterior palatine canal. V.—Vomer. M.P.—Maxillary palate. P.F.—Palatine fissure.

*Scheme III.*

*Complete alveolar cleft double—cleft between lateral incisors and canine; compare with lion.*

*Complete double alveolar cleft—cleft between incisors.*

*Unilateral cleft between central and lateral incisors.*

I.—Incisors. P.F.—Premaxillary fissure. C.—Canine. M.P.—Maxillary palate. V.—Vomer. O.S.—Os incisivum.

Fig. 10.

the premaxilla may regain its former importance: it may once more appear on the face, in the roof of the mouth, and actually bear teeth; and when it does so, it prevents the superior maxilla reaching the middle line, and between it and the maxilla is a gap—the real premaxillary fissure—wide open, and *that is alveolar cleft palate*. The more completely the premaxilla gains its former importance, the more complete is the attempt at prognathism, and we recognise the condition as double alveolar cleft palate. The incisor teeth are now borne by the premaxillæ (os incisivum), and are separated by the alveolar cleft from the canine and molars, which are contained in the maxilla, just as they are in the lion. If the premaxilla of one side develop enough to regain a single incisor tooth, then the gap is between the first incisor and the second incisor, the latter tooth being lodged in the maxilla. Alveolar cleft palate, then, is the result of an attempt to restore the prognathous condition of our remote ancestors.

Let us now contrast this statement with the accepted theories as to the causation of alveolar cleft palate.

Goethe, in 1780, regarding the palatine fissure above mentioned as the premaxillary fissure and the bone in front of it as the premaxilla, as it bore incisor teeth, said that alveolar cleft palate was the result of non-obliteration of this fissure, and that normally this fissure in the early months of fœtal life was present on the face. Callender, in 1869, however, debating this, denied that the fissure was present on the face, because it was hidden by a down growth of the incisor process of the maxilla. But Albrecht, in 1879, and later in many papers, pointed out that the cleft was not always between the canine and the lateral incisor teeth, but that it was often between the two incisors themselves; and he, to account for this, described a premaxilla as ossifying in two pieces, one internal to the cleft, the other external, and to these pieces he applied the term endognathion to that piece containing the central incisor, and mesognathion to that in which the lateral incisor is embedded. He likewise was of the opinion that the palatine fissure was the premaxillary, and that that branch of it which partially separates under ordinary circumstances the bone containing the central incisor from the lateral had extended through the alveolar wall, which was supposed to be entirely premaxillary. There still remained the difficulty of this fissure not being normally seen on the face in early development, which was got over by the supposition that in front of the endognathion and mesognathion a lamina grew down from the incisor process and covered up the fissure at an early period of fœtal life.

It is hoped that it has been clearly demonstrated that the incisor teeth are not normally contained in the premaxilla; in fact, that the true premaxilla has altogether escaped notice as such, and as a consequence the accepted ideas as to the premaxillary fissures are unwarranted. The premaxilla of man corresponds, both by its position, its relations to the anterior palatine canal, its articulation and its development, with that part of the premaxilla in animals which has been described as the palatine process. By force of circumstances it has lost its alveolo-facial part, the part which should have the incisor teeth which is present in lower animals, and which has been supplanted by the forward growth of the two maxillæ, and the premaxilla of man only reappears in its proper role growing its alveolo-facial part and its incisor teeth in alveolar cleft palate.

In conclusion, it may be stated that this premaxilla is no new discovery, for as early as 1864 Rambaud and Renault described it as to its topographical position accurately and in detail by the name of *os sous-vômerien;* but they did not realise its true morphological importance, for they simply regarded it as an additional centre of ossification of the superior maxilla.

## Figures and Tables

**Fig. 1. f1:**
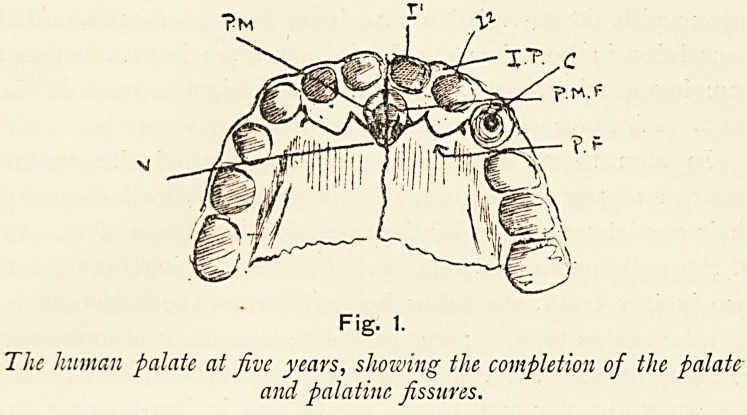


**Fig. 2. f2:**
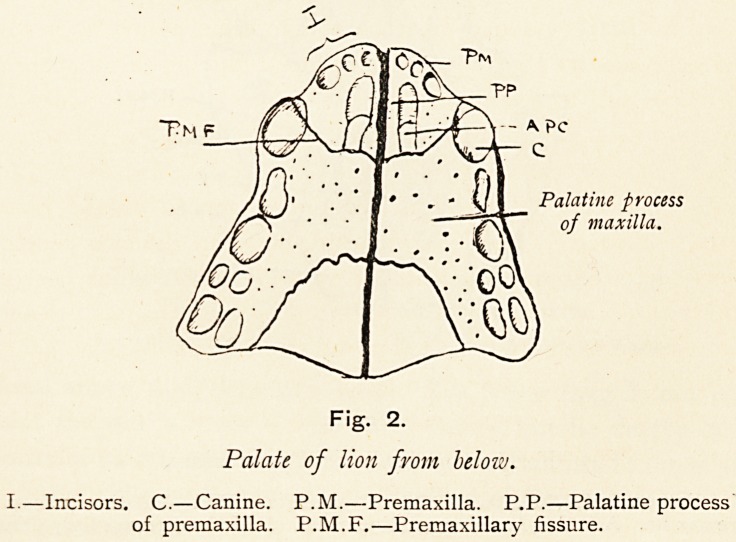


**Fig. 3. f3:**
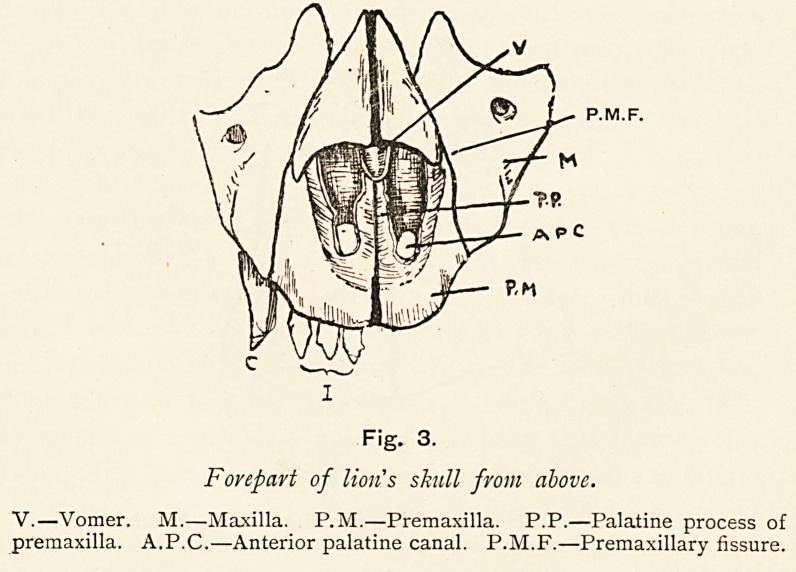


**Fig. 4. f4:**
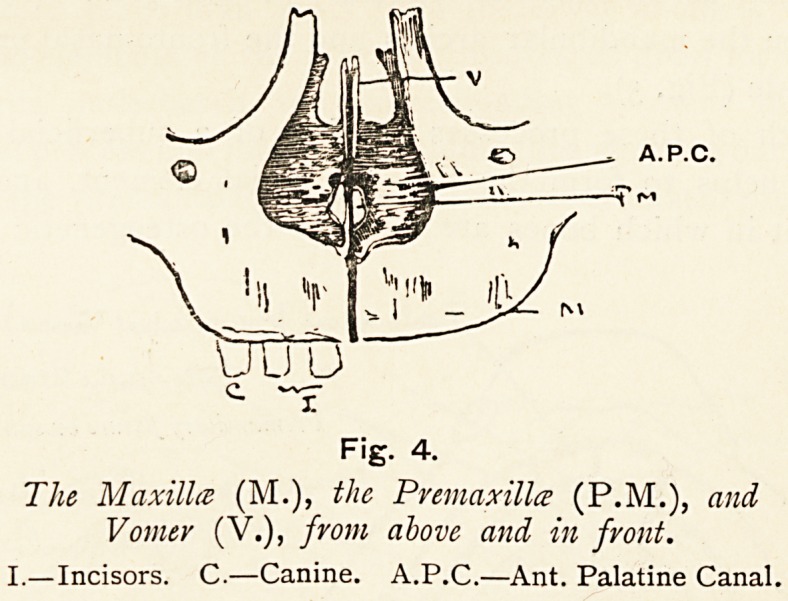


**Fig. 5. f5:**
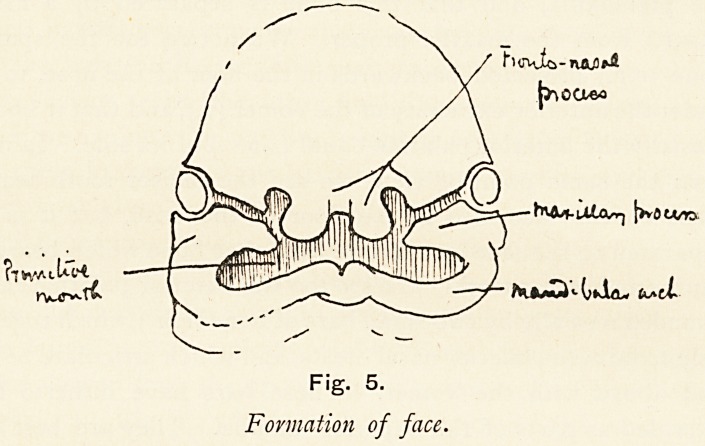


**Fig. 6. f6:**
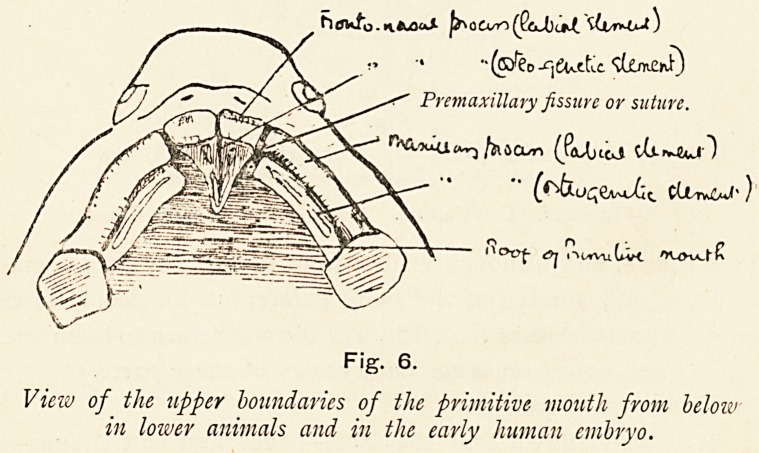


**Fig. 7. f7:**
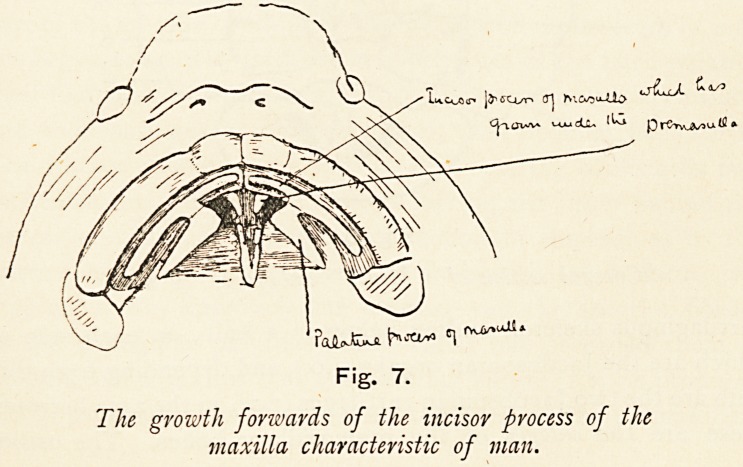


**Fig. 8. f8:**
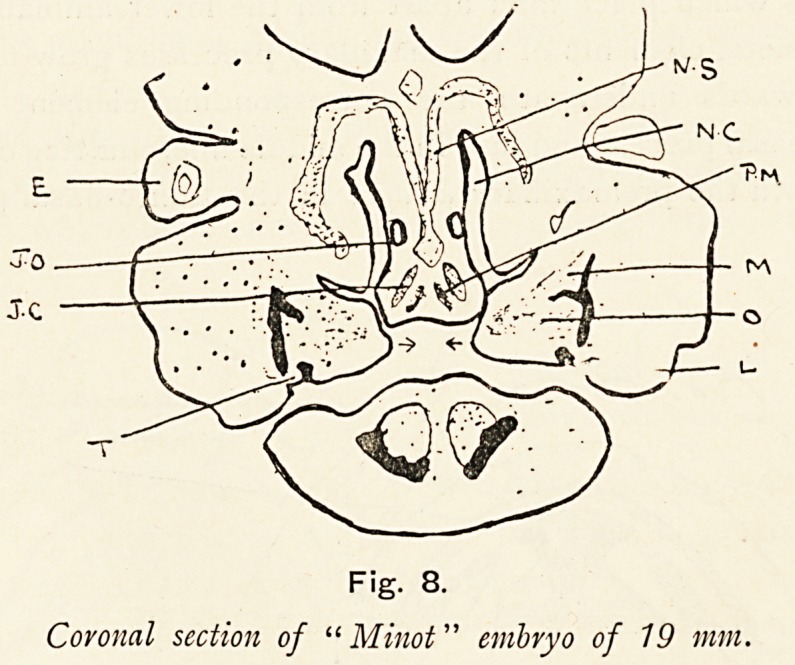


**Fig. 9. f9:**
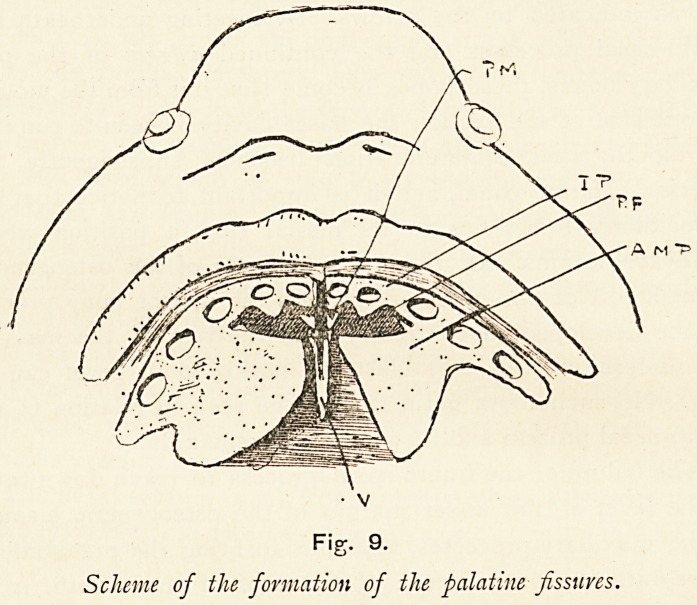


**Fig. 10. f10:**